# A Review of Biological and Sustainable Management Approaches for *Alphitobius diaperinus*, a Major Pest in Poultry Facilities

**DOI:** 10.3390/vetsci12020158

**Published:** 2025-02-12

**Authors:** Ozge Tufan-Cetin, Huseyin Cetin

**Affiliations:** 1 Department of Environmental Protection Technology, Vocational School of Technical Sciences, Akdeniz University, 07070 Antalya, Türkiye; 2 Faculty of Science, Department of Biology, Akdeniz University, 07070 Antalya, Türkiye

**Keywords:** *Alphitobius*, biological control, entomopathogens, integrated pest management, poultry

## Abstract

The lesser mealworm (*Alphitobius diaperinus*) is a significant pest in poultry facilities, transmitting pathogens such as *Salmonella* and causing structural damage in poultry operations. Challenges such as insecticide resistance and environmental concerns associated with the widespread use of traditional insecticides underscore the need for sustainable alternatives. This review examines biological and sustainable management strategies for managing the lesser mealworm, including entomopathogenic fungi, bacteria, nematodes, plant essential oils-extracts, pheromones, and diatomaceous earth. These eco-friendly approaches highlight fungi and nematodes for their effectiveness in larval control and essential oils for their insecticidal and repellent properties. The application of pheromones and diatomaceous earth further complements other methods within integrated pest management (IPM) systems. Integrating these biological tools into IPM offers a sustainable and effective solution for reducing *A. diaperinus* populations, addressing both health and environmental concerns.

## Simple Summary

The lesser mealworm (*Alphitobius diaperinus*) is a major pest in poultry facilities, causing health and economic issues by spreading pathogens and damaging building structures. The heavy use of chemical insecticides to control this pest has led to resistance, health concerns, and residue problems in poultry products. This review examines eco-friendly biological control methods, such as entomopathogenic fungi, bacteria, and nematodes, as well as sustainable management strategies, including plant essential oils, extracts, pheromones, and diatomaceous earth. These methods offer sustainable alternatives for managing lesser mealworm populations and are valuable tools in integrated pest management strategies, promoting both animal health and environmental safety.

## 1. Introduction

Lesser mealworm, small *mealworm* (*Alphitobius diaperinus* (Panzer, 1797) (Coleoptera: Tenebrionidae)), is a pest of stored food grain products and poultry farming facilities. It is thought to have originated in sub-Saharan Africa and has spread worldwide. Adults are shiny black in colour, 6 mm long, and broadly oval in shape. Larvae develop six to 11 instars, reaching a length of up to 11 mm in the last instar. Larvae and adults are mostly nocturnal, active at dusk, and under litter is their favorite habitat [1,2].

Although it can be used as human food, *A. diaperinus* is a well-documented vector for several pathogens, including bacteria, viruses, fungi, and parasites, which significantly affect the health and food safety of poultry [3,4]. In a study, the role of beetles as intermediate hosts for *Raillietina cesticillus*, a poultry tapeworm, was highlighted. The research found that nearly 30% of beetles collected from infected farms carried the parasite, contributing to the persistence of tapeworm infections in poultry houses [5]. Similarly, *A. diaperinus* larvae have been shown to efficiently transmit *Salmonella typhimurium* to broiler chickens, spreading the pathogen through both direct contact and contaminated feed, with transmission persisting for weeks [6]. In a further study, it was demonstrated that *A. diaperinus* can carry multidrug-resistant *Salmonella enterica* strains, making the beetle a significant vector in spreading zoonotic pathogens that are resistant to antibiotics, complicating efforts to control infections in poultry environments [7]. In addition, Crippen et al. found that *A. diaperinus* plays a crucial role in the horizontal transfer of *Salmonella* within poultry facilities, as these beetles can acquire the bacteria from contaminated manure and spread it across flock rotations, perpetuating the pathogen’s presence in the environment [8].

Beyond its role in transmitting bacterial and parasitic pathogens, *A. diaperinus* has been implicated in the spread of toxigenic fungi like *Aspergillus flavus*, which can lead to food contamination and health risks due to mycotoxin production. This dual role, as both a carrier of parasites and a contributor to fungal contamination, presents a complex challenge for food safety [9]. Finally, Huber et al. [10] investigated the beetle’s ability to carry *Histomonas meleagridis*, a protozoan that causes histomoniasis in poultry. While *A. diaperinus* larvae were able to harbor the parasite, the study concluded that they are unlikely to serve as a major vector for its transmission between flocks. Apart from being a vector, this insect is known to be pecked by chickens and ingested into their crops (craws), where it can remain. Upon slaughter, the insect may still be present in the crop, making the meat unsuitable for sale and rendering the product unmarketable [11].

**Figure 1:**
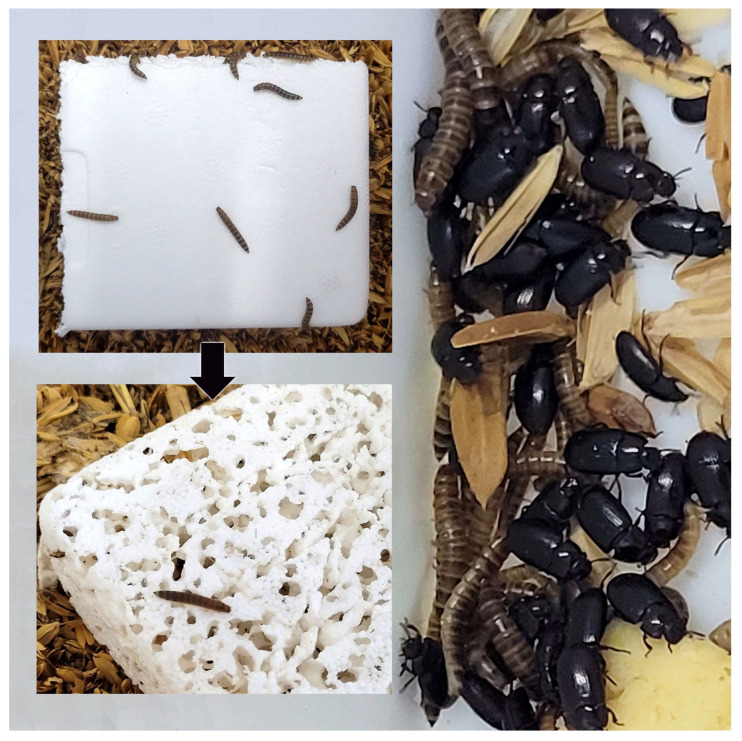
Damage caused by larvae of *Alphitobius diaperinus* to polyurethane material used as construction material under laboratory conditions and general appearance of adults and larvae of the species (Photos by Huseyin Cetin).

In addition to its role in disease transmission, *A. diaperinus* causes significant damage to poultry house structures, especially insulation materials. A study investigating the capacity of these insects to tunnel through and damage insulation materials such as polystyrene, polyurethane, and fiberglass, revealed that polystyrene was particularly susceptible to larval tunneling and pupation (Figure 1) [12]. This damage not only compromises the structural integrity of poultry houses but also reduces thermal efficiency, leading to increased energy costs and the need for frequent maintenance. Further research has shown how the beetles’ dispersal behavior is influenced by environmental factors such as manure moisture levels and construction materials [13]. It found that larvae and adults were more likely to disperse from manure when moisture levels increased, and that wooden surfaces allowed the beetles to climb higher than concrete surfaces. This suggests that poultry houses with wooden pit walls and support beams are more prone to insulation damage compared to those with concrete foundations. Thus, *A. diaperinus* poses the dual threat of being a vector of numerous pathogens, antimicrobial-resistant bacteria, parasites and fungi, as well as causing structural damage to buildings through degradation of insulation materials. Effective pest control strategies are essential to mitigate both the health risks, and the economic damage associated with these beetles [14].

The lesser mealworm is commonly controlled in poultry operations using chemical insecticides. Various chemicals with different modes of action are applied in poultry houses; however, many of these chemicals pose risks to both animal and human health, as well as causing residue problems. Additionally, many studies have shown that these insects have developed resistance to several groups of insecticides, making pest control increasingly difficult and emphasizing the need for alternative management [15,16,17].

A study conducted to examine the susceptibility of *A. diaperinus* to bifenthrin, imidacloprid and spinosad revealed that insect populations in Georgia show different levels of resistance to these insecticides. A study conducted to examine the susceptibility of *A. diaperinus* to bifenthrin, imidacloprid, and spinosad revealed that insect populations in Georgia exhibited different levels of resistance to these insecticides [18]. The results showed extremely high resistance to imidacloprid, with resistance levels exceeding 3000-fold, while lower resistance was observed against bifenthrin and spinosad. This finding indicates that imidacloprid may no longer be an effective control method in certain regions, highlighting the need for alternative strategies to manage *A. diaperinus* populations. Similarly, Hamm et al. [19] studied the resistance of *A. diaperinus* to cyfluthrin and tetrachlorvinphos, two commonly used insecticides in poultry farms in the eastern United States. The results revealed varying levels of resistance across different populations, with cyfluthrin resistance ratios reaching up to 29-fold in larvae and 9.5-fold in adults. Resistance to tetrachlorvinphos was even more pronounced, with some individuals surviving exposure to doses 1000 times higher than those required to kill susceptible strains. These findings underscore the widespread nature of insecticide resistance and the urgent need for integrated pest management (IPM) strategies, including insecticide rotation and the use of biological control agents, to combat this growing problem.

In another study, the susceptibility to various insecticides of *A. diaperinus* populations collected from six broiler facilities in eastern Texas was examined [20]. The results indicated that, while the beetles showed some resistance to products like Tempo SC Ultra and Talstar WP, newer generation pyrethroids were more effective in controlling the populations. However, a significant recovery rate of 50–60% was observed in populations treated with Dragnet SFR, suggesting that some insecticides are less effective in controlling resistant populations. Hickmann et al. [17] investigated the resistance of *A. diaperinus* populations from southern Brazil to two commonly used insecticides, cypermethrin and chlorpyrifos. The study found significant geographical variation in resistance levels, with populations from Paraná state showing resistance up to 31.2-fold for chlorpyrifos. These findings highlight the widespread nature of insecticide resistance and the need for insecticide resistance management strategies. The researchers recommend implementing diagnostic concentrations of 15 µg/cm^2^ for cypermethrin and 12 µg/cm^2^ for chlorpyrifos to monitor resistance development effectively.

Pesticides used in poultry farming not only lead to the development of resistance in target organisms but also leave residues in the meat and eggs of livestock and pose a risk of contaminating the environment through farm waste. Studies on this issue point to serious health and environmental risks. For instance, one study detected widespread DDT residues in chicken and duck eggs sampled across China, with these residues attributed to local industrial activities or illegal pesticide usage [21]. Other research showed that some pesticide metabolites in poultry droppings could be detected for at least 30 days, highlighting the impact of environmental conditions on the persistence of these residues [22]. Wang et al. [23] revealed that the pesticide fipronil quickly accumulates in eggs and edible tissues, particularly in the skin and yolk, and that these residues transform into more toxic metabolites, thereby increasing health risks. Furthermore, a comprehensive review emphasized the significant environmental footprint of poultry farming and highlighted that poultry litter and waste can contain pesticide residues and other pollutants, which contaminate soil, air, and water, posing serious threats to environmental and human health [24]. All these studies have shown that overuse of chemical insecticides leads to increased resistance in *A. diaperinus* populations. This resistance not only reduces the effectiveness of insecticides but also complicates pest control in poultry facilities. Therefore, adopting IPM strategies, including insecticide rotation and the use of biological control methods, is essential to mitigate the health and structural threats posed by *A. diaperinus*.

Biologic and sustainable pest management significantly reduces reliance on chemical pesticides, thereby minimizing environmental pollution and health risks. These approaches effectively mitigate pest resistance to chemicals and foster long-term ecological balance. Nanoparticle-based delivery systems, RNA interference technology, symbionts, insect gut microbiome modifications, and transgenic resistance present significant opportunities for developing innovative strategies [25].

The successful implementation of biological control requires meeting specific criteria, including the mass production of bio-agents, proper training for practitioners, and regulatory support to ensure accessibility and effectiveness [26]. Conservation strategies, such as creating habitats to support natural enemies, are essential for sustaining these systems [27]. Despite challenges like high initial costs and limited farmer knowledge, advancements in technology hold promise for optimizing biological control [28]. These innovations underscore the growing potential of biological control as a sustainable and effective alternative to chemical pesticides.

## 2. Materials and Methods

This review was conducted by searching various databases including Web of Science, Science Direct, Google Scholar and PubMed. We focused on articles discussing biological control strategies and sustainable management approaches to manage *A. diaperinus* in poultry environments using keywords such as “entomopathogenic fungi (EF)”, “entomopathogenic bacteria (EB)”, “entomopathogenic nematodes (EN)”, “plant essential oils (PEO)”, “plant extracts (PE)”, “pheromones (P)”, “diatomaceous earth (DE)” and/or “*Alphitobius diaperinus*”, “biological control”, “poultry”, “management strategies”, “integrated pest management”. Successful biological control methods applied to *A. diaperinus* are explained under the main headings of entomopathogen nematodes, entomopathogen bacteria, entomopathogen fungi, plant essential oils and extracts, pheromones and diatomaceous earth.

## 3. Results

### 3.1. Biological Control Strategies of A. diaperinus

#### 3.1.1. Entomopathogenic Fungi

Entomopathogenic fungi (EF) are microorganisms that naturally infect and kill insect hosts, playing a crucial role in biological pest control. These fungi, primarily from genera such as *Beauveria, Isaria*, *Lecanicillium*, *Metarhizium*, *Paecilomyces*, have been studied for their effectiveness against a variety of insect pests in agriculture and poultry [29,30,31].

Studies have demonstrated the lethal effects of EF on various insect pests, highlighting their potential as eco-friendly biocontrol agents. Zibaee et al. [32] showed *that Beauveria bassiana* produces secondary metabolites that suppress immune responses in *Eurygaster integriceps* and reduce hemocyte counts, leading to mortality. Similarly, *Isaria fumosorosea* caused up to 91% mortality in the larvae of *Plutella xylostella* within six days by producing toxic metabolites [33]. Many such findings underline the diverse applications of EF in sustainable pest management and offer alternatives to chemical pesticides [34,35,36].

##### Mechanism of Action of EF Controlling *A. diaperinus*

Unlike entomopathogenic nematodes (EN) and entomopathogenic bacteria (EB), which require ingestion by the host to establish infection, certain entomopathogenic fungi are capable of directly invading their insect hosts through the cuticle, the outer protective layer of the exoskeleton [37]. This adaptation enables these fungi to bypass the need for oral uptake and directly exploit their hosts, a feature that makes them highly effective biological control agents in various ecosystems.

The infection process is initiated when fungal spores, known as conidia, attach to the insect’s cuticle. This attachment is facilitated by hydrophobic and electrostatic interactions, as well as specialized adhesive proteins on the spore surface [38]. Upon successful adhesion, the spores germinate under favorable environmental conditions, including adequate humidity and temperatures typically ranging between 20 °C and 30 °C [39]. Germination involves the production of a germ tube, which penetrates the cuticle using a combination of enzymatic and mechanical mechanisms. Enzymes such as proteases, lipases, and chitinases degrade the structural components of the cuticle, while mechanical pressure from the growing fungal structures aids in penetration [40,41].

Once inside the insect’s body cavity, known as the hemocoel, the fungus proliferates and colonizes the internal tissues. This growth is accompanied by the secretion of toxic secondary metabolites, such as destruxins and beauvericin, which suppress the insect’s immune defenses and disrupt physiological processes [42]. These toxins contribute to systemic infections that compromise vital biological functions, leading to the insect’s death within days.

Following the death of the host, the fungus sporulates on the cadaver, emerging as a dense mat of hyphae that produces new conidia. These conidia are released into the environment, where they remain viable for extended periods, ready to infect new hosts upon contact [43]. This ability to produce infectious propagules ensures the continuation of the infection cycle and highlights the potential of these fungi in sustainable pest management strategies.

##### Studies on the Control of *A. diaperinus* with EF

Studies on *A. diaperinus* using EF focus on exploring their potential as biological control agents to manage this pest, which poses significant challenges in poultry production due to its role as a vector of pathogens and its resistance to chemical treatments. Chernaki-Leffer et al. [44] evaluated the pathogenicity of various EF, including *Beauveria bassiana*, *Paecilomyces* spp., and *Metarhizium anisopliae*, against the lesser mealworm. The study showed that *M. anisopliae* isolates CNPSo-Ma352 and CNPSo-Ma356 were highly effective against *A. diaperinus* larvae, with LD_50_ values ranging from 2.2 × 10^4^ to 4.5 × 10^4^ conidia per larva. However, adult beetles were more resistant to fungal infection, with LD_50_ values of 1.3 × 10⁵ to 2.1 × 10⁵ conidia per adult. This difference in susceptibility suggests that while larvae are more vulnerable to fungal infection, additional control methods may be necessary for effective management of adult populations.

A study conducted in Poland investigated local strains of *B. bassiana*, *M. anisopliae*, *Isaria fumosorosea*, and *I. farinosa* for their potential as biological control agents against *A. diaperinus*. The researchers confirmed that larvae are significantly more susceptible to fungal infection than adults. While adult beetles showed low susceptibility, with mortality rates of 36% for *M. anisopliae* and 26% for *B. bassiana*, larvae exhibited 100% mortality in most isolates, except for *I. fumosorosea*. The strain *B. bassiana* 3K showed particularly high larval mortality and demonstrated fungal overgrowth on dead larvae, indicating its potential as a biocontrol agent in poultry farms [45].

Santoro et al. [46] examined 30 isolates of *B. bassiana* to identify the most effective strains for controlling lesser mealworm populations in poultry farms. Four isolates—CG 71, CG 152, UNIOESTE 4, and UNIOESTE 40—achieved more than 40% mortality within 10 days. The most virulent isolate, UNIOESTE 4, had an LC_50_ of 0.8 × 10^6^ conidia/mL, making it the most promising candidate for further study. Genetic analysis confirmed that UNIOESTE 4 belongs to *B. bassiana* Clade A, suggesting that specific fungal strains within *B. bassiana* could offer targeted and effective pest control in IPM programs.

A study further demonstrated the effectiveness of *B. bassiana* and *M. anisopliae* against lesser mealworms in laboratory tests. The *M. anisopliae* K isolate was the most effective, achieving 80–90% mortality in mature larvae within 14 days [47]. The residual activity of fungal conidia remained potent for up to 14 days after application, suggesting that *M. anisopliae* could provide long-term pest control in poultry houses. This prolonged residual effect is particularly beneficial in environments like poultry farms, where repeated applications are difficult.

Rice et al. [48] evaluated granular formulations of *B. bassiana* and *M. anisopliae* in commercial broiler houses. Over five production batches, *B. bassiana* suppressed *A. diaperinus* populations by 72%, while *M. anisopliae* achieved a 50% reduction, both outperforming standard chemical insecticides, which achieved 48% suppression. These results demonstrate that fungal-based biopesticides can be as effective as chemical insecticides, offering a more sustainable pest control option. However, the study also noted that the fungicide imazalil, applied during one batch, interfered with fungal efficacy, highlighting the importance of avoiding fungicides in conjunction with EF treatments.

##### Synergistic Effect Studies with EF on *A. diaperinus* Control

In addition to direct fungal applications, combining fungi with other control agents can enhance their efficacy. Rice and Furlong [49] explored the synergistic effects of combining *B. bassiana* with insecticides such as β-cyfluthrin, imidacloprid, and spinosad. The combination of β-cyfluthrin with *B. bassiana* significantly increased mortality and conidiogenesis rates in larvae. These findings suggest that sublethal concentrations of insecticides could be used alongside fungal treatments to enhance pest control efficacy and reduce the overall amount of chemicals required, offering a more sustainable pest management approach.

The insecticidal potential of fungal extracts from *B. bassiana* strains CG71 and UNI40 was investigated. Methanolic extracts from the UNI40 strain achieved 95.97% mortality in adult lesser mealworms, whereas the CG71 methanolic extract resulted in 69.23% mortality [50]. These extracts contained various metabolites, including Sumiki’s acid, dipicolinic acid, and cyclodepsipeptides, which likely contributed to their insecticidal properties. This research suggests that fungal extracts, in addition to direct fungal applications, could serve as potent biocontrol agents for managing lesser mealworm populations.

The effectiveness of EF can be influenced by environmental conditions, such as temperature and the presence of poultry litter. Alexandre et al. [51] explored the impact of these factors on the germination, growth, and virulence of *B. bassiana* and *M. anisopliae* isolates. At 26 °C, *B. bassiana* isolates showed higher conidial production (up to 11 × 10^8^ conidia/larval cadaver) compared to *M. anisopliae*. However, at 32 °C, both fungi experienced reduced virulence, with larvae being more sensitive to fungal infection at the lower temperature. The data from this study align with our observations, indicating that while chicks require higher temperature levels, adult chickens have lower temperature needs. Chicks need a temperature of 32–35 °C during the first week, which is gradually reduced each week, reaching 22–24 °C by the 6th week. Although these temperature levels and appropriate humidity conditions in poultry houses are suitable for the development oof EF, we suggest that applying EF 1–2 weeks after the chicks are introduced into the poultry house could achieve more effective insect control. Additionally, poultry litter impacted fungal efficacy, with both new and used litter reducing the overall effectiveness of the fungi. This study highlights the importance of considering environmental factors when applying EF in poultry facilities.

#### 3.1.2. Entomopathogenic Bacteria

Entomopathogenic bacteria (EB) are microorganisms that infect and kill insect hosts. *Bacillus*, *Serratia*, *Pseudomonas*, and *Photorhabdus* are the most widely studied genera [52,53]. Among them, *Bacillus thuringiensis* (*Bt*) is the best known and commercially used EB. These bacteria naturally exist in soil and are pathogenic to a wide range of insect pests.

Different species of *Bacillus* target specific insect groups. For example, *Bt israelensis* is highly effective against mosquitoes (Culicidae) and black flies (Simuliidae), particularly their larvae [54], while *Bacillus sphaericus* is also used for mosquito control, especially in polluted water environments [55]. *Bt* subsp. *kurstaki* is primarily used to control leaf-feeding caterpillars (Lepidoptera), such as the cabbage worm (*Pieris rapae*) [56], corn borer (*Ostrinia nubilalis*) [57,58], and codling moth (*Cydia pomonella*) [59]. When ingested, *Btk* toxins damage the gut cells of caterpillars, leading to their rapid death. *Bt* has been used extensively to control lepidopteran larvae, such as the European corn borer (*O. nubilalis*) [60] and the diamondback moth (*Plutella xylostella*) [61]. Other strains of *Bt* are effective against coleopteran pests like the Colorado potato beetle (*Leptinotarsa decemlineata*) [62] and dipteran pests like mosquitoes (*Aedes aegypti*) [63]. *Photorhabdus* and *Xenorhabdus* species, symbiotic with EN, are particularly effective against soil-dwelling insects like weevils [64] and rootworms [65]. Other species, like *Bacillus subtilis*, are commonly used in plant pathogen control, indirectly reducing pest pressures by strengthening plant defenses or inhibiting plant diseases [66,67].

##### Mechanism of Action of EB Controlling *A. diaperinus*

The mode of action of EB involves the production of toxins, which either directly attack the insect’s gut cells or disrupt its immune system. For example, *Bt* produces crystalline (Cry) and cytolytic proteins [68]. When ingested by insects, these proteins bind to receptors in the insect’s gut epithelial cells, causing cell lysis, disruption of the gut lining, and ultimately septicemia, leading to death. Other bacteria, such as *Photorhabdus* and *Xenorhabdus*, are symbiotic with nematodes and are delivered into insect hosts when nematodes invade their bodies [69,70]. Once inside, these bacteria produce various virulence factors, including toxins and enzymes, that suppress the host’s immune system and rapidly cause death through septicemia [71].

##### Studies on the Control of *A. diaperinus* with EB

Studies on the effectiveness of EB against the lesser mealworm have produced mixed results. *Bt* strains, particularly the Cry3Bb toxin, have shown potential, but their efficacy varies depending on the experimental conditions and concentrations used. For example, Hua et al. [72] reported that *A. diaperinus* larvae possess receptors for the Cry3Bb toxin expressed by *Bt*, indicating that the bacterium has potential as a control agent. Also, earlier studies by Maciel [73] also showed that approximately 26% of fourth instar litter beetles were killed within 7–9 days after exposure to non-commercial *Bt* variants. On the other hand, Koc et al. [74] and Berretta et al. [75] found that *Bt* strains were not effective at the tested concentrations for controlling *A. diaperinus* larvae. This result was unexpected, especially given the existence of patents Hickle et al. [76] suggesting that these *Bt* variants could be effective against this species. Interestingly, Hasan et al. [77] reported that prolonged exposure to a *Bt* subsp. *kurstaki*-treated diet reduced lesser mealworm populations under field conditions. Their experiment revealed that first to eighth instar larvae failed to pupate when exposed to a 300 ppm *Bt* concentration. However, Koc et al. [74] observed different results, even with a higher concentration of 500 ppm, where no significant impact on pupation or emergence rates was recorded. Despite a 17% mortality rate in fifth instar larvae, about 70% successfully reached adulthood, showing variability in the efficacy of *Bt*-based products against *A. diaperinus* under different conditions. In another study, it showed low toxicity against *Bacillus toyonensis* biovar *thuringiensis* on *A. diaperinus* [78].

##### Synergistic Effect Studies with EB on *A. diaperinus* Control

Park et al. [79] explored ways to enhance the toxicity of *Bt* toxins against *A. diaperinus* larvae by using coleopteran cadherin fragments. The study focused on Cry3Aa, Cry3Bb, and Cry8Ca insecticidal proteins produced by different strains of *Bt*, which are commonly used to control coleopteran larvae. The researchers found that cadherins, proteins located in the insect midgut epithelium, act as receptors for Cry toxins. By adding a specific truncated cadherin fragment (DvCad1-CR8–10) from the western corn rootworm (*Diabrotica virgifera virgifera*), the toxicity of the Cry3Aa, Cry3Bb, and Cry8Ca toxins to *A. diaperinus* larvae increased by 3- to 5.9-fold. This effect was attributed to the cadherin fragment’s ability to bind Cry toxins with high affinity and promote oligomerization, enhancing the efficacy of the toxins. The study concludes that using cadherin fragments as synergists for Cry proteins offers a promising strategy to improve the biological control of *A. diaperinus* in poultry facilities, where chemical control is often inadequate due to insecticide resistance.

#### 3.1.3. Entomopathogenic Nematodes

Entomopathogenic nematodes (EN) are widely used biological agents in pest management. The first discoveries of entomopathogenic nematodes came with the demonstration of pathogenicity of species in the families Steinernematidae and Heterorhabditidae against insect pests [80,81,82]. After that, researchers observed that nematodes released bacteria (e.g., *Xenorhabdus* sp. and *Photorhabdus* sp.) into their insect hosts, which produced toxins that killed the insects [83,84]. Important species such as *Steinernema carpocapsae* were recognized for their pest control potential, and commercial interest increased as advances in IPM prioritized environmentally friendly alternatives to pesticides. To date, approximately 120 species of entomopathogenic nematodes have been identified [85]. They are used effectively in controlling both agricultural and forest pests. In agriculture, they have been shown to be effective against pests such as aphids [86], root nematodes [87], thrips [88], and whiteflies [89]. In forestry, their use is becoming more widespread for controlling pests like the pine processionary moth (*Thaumetopoea pityocampa*) [90].

EN are prepared for application using two main methods. Commercial products are typically sold in water-based suspensions or powder formulations, which are mixed with water before being applied to agricultural areas. They are applied to soil or plant surfaces using irrigation systems, backpack sprayers, or other spray devices. Maintaining low temperatures and moisture during application is critical to ensuring the nematodes remain viable. Another method involves sourcing nematodes from natural environments. In laboratory settings, nematodes are propagated using host insects, commonly *Galleria mellonella* (greater wax moth larvae). After infecting these insects, the nematodes proliferate within the dead host tissues and can be isolated. When isolating nematodes from agricultural fields, dead insects are examined to determine whether entomopathogenic nematodes are present in their bodies. This method allows for the use of nematodes that are naturally found in the area, making them more effective in local pest control efforts [91].

##### Mechanism of Action of EN Controlling *A. diaperinus*

EN can kill their hosts through two main mechanisms: one that relies on symbiotic bacteria and another that kills directly. Many species release symbiotic bacteria into the host’s body, which spread throughout the tissues and cause a toxic effect, leading to the insect’s death [70]. In contrast, some nematode species kill their hosts without the aid of bacteria by mechanically damaging the host’s tissues or depleting essential nutrients [92].

These parasitic worms thrive and move most effectively in moist environments, as they rely on moisture to maintain their viability. They enter insect hosts through natural openings such as the mouth, anus, or tracheal system, or by directly penetrating the cuticle. The nematode life cycle begins when they invade the host and release symbiotic bacteria into the host’s tissues. Once the host is killed, the nematodes reproduce inside the dead tissues, and the new generation disperses into the surrounding environment. This cycle is more efficient in moist soils or areas rich in organic matter. A significant advantage of entomopathogenic nematodes is that they are environmentally friendly and do not leave harmful residues. Additionally, the likelihood of insects developing resistance to these biological agents is very low, almost impossible. Typically, once enough nematodes invade the host, death occurs within a few days. After killing the target organism, nematodes can reproduce and infect other pests in moist environments [93,94].

##### Studies on the Control of *A. diaperinus* with EN

EN have also been successfully used to control the lesser mealworm [95,96,97]. Studies have shown that nematodes exhibit higher lethal effects on larvae compared to adult insects [95,98]. After entering the insect’s body, the nematodes colonize the tissues, typically causing death within 48 to 72 h. Over time, the mortality rate increases, and larvae are more susceptible to nematode infections than adults. In this context, *Heterorhabditis bacteriophora* [99], *S. carpocapsae* [94,100] and *S. feltiae* [93,100,101] are the most studied and effective nematode species for controlling lesser mealworms. In addition, other researchers showed that *S. affine*, *S. affinis*, *S. carpocapsae*, *S. feltiae*, *H. riobrave*, *H. indica*, *H. bacteriophora* were effective nematodes towards the lesser mealworms [81,102]. These nematodes exhibit greater lethal effects on larvae compared to adults, making them particularly suitable for larval control. Koc et al. [97] illustrated this vulnerability in their study, where *S. carpocapsae* was tested against different lesser mealworm populations. The LC_50_ values for larvae ranged from 31.2 to 70.9 infective juveniles (IJs) per milliliter (mL), while adult beetles required significantly higher concentrations, with LC_50_ values between 85.9 and 418.8 IJs/mL. These findings highlight the structural and behavioral differences that make larvae more susceptible to nematode infection compared to adult beetles. This difference is attributed to several factors, including the larvae’s more segmented and flexible body structure, thinner cuticle, and increased mobility. In contrast, adult beetles possess a thicker, more rigid cuticle and fewer body segments, making them more resistant to nematode penetration. Larvae of *A. diaperinus* exhibit a highly segmented body with a thinner cuticle compared to adults, which significantly enhances their susceptibility to nematode infection. The segmented body of larvae provides more surface area and flexibility, allowing nematodes more entry points to invade the insect. Geden et al. [103] raised larvae in sandy loam and clay soils and thinned their cuticles. Long-term control was achieved because the larvae were more susceptible to the nematode *S. feltiae*. Early larvae and pupae were particularly vulnerable to *Heterorhabditis heliothidis*, while *S. glaseri* was more effective against adult insects, but was still limited by adult insect resistance mechanisms.

Additionally, larvae are much more active than adults, moving frequently through the litter, which increases their likelihood of encountering nematodes. This heightened mobility places them in areas where nematodes are active, improving the chances of successful infection. Alves et al. [104] demonstrated that *Steinernema arenarium* was highly effective against larvae, achieving up to 99% mortality, but its effectiveness dropped significantly when targeting adults due to their lower exposure and thicker cuticle. The study also found that high temperatures and poultry litter further reduced the nematodes’ efficacy, indicating that environmental conditions play a critical role in nematode success.

A study on various *Steinernema* species, including *S. carpocapsae*, *S. feltiae*, and *S. scapterisci*, revealed that adult beetles exhibited lower susceptibility across all tested strains [100]. The LC_50_ values ranged from 1.5 to 77.0 nematodes per beetle in filter paper assays and 5.8 to 14.6 nematodes per beetle in poultry litter assays. These results indicated that adult beetles are significantly more resistant due to their thicker cuticle and fewer segments, making infection more difficult for nematodes. In a study beetles collected from six poultry farms in Türkiye were bred and tested with *S. feltiae* nematodes at concentrations of 25–200 infective juveniles (IJs)/mL, revealing higher susceptibility in larvae (LC_50_: 33.17–83.12 IJs/mL) compared to adults over a five-day mortality assessment [11].

##### Synergistic Effect Studies with EN on *A. diaperinus* Control

While nematodes face challenges in infecting adult beetles, combining them with other biological agents or using novel technologies can enhance their efficacy. Kucharska et al. [95,98] explored the combination of nematodes with nanoparticles (silver, gold, and copper) and found that the addition of nanoparticles generally increased nematode effectiveness, particularly against larvae. However, gold nanoparticles negatively affected adult beetle mortality when combined with *S. feltiae*. These results suggest that, while larvae remain the primary target, synergistic approaches may help improve nematode performance against adults.

### 3.2. Sustainable Control Approaches of A. diaperinus

#### 3.2.1. Plant Essential Oils and Extracts

Plant essential oils (PEO) and extracts (PE) have become increasingly popular in pest management due to their eco-friendly, multi-faceted modes of action and low risk of resistance development. These natural products contain bioactive compounds such as terpenes, phenols, aldehydes, and others that contribute to their broad-spectrum insecticidal, repellent, and antimicrobial effects. PEO and PE are particularly valuable in IPM because they do not leave harmful chemical residues, making them a safer alternative to conventional pesticides. Their versatility allows them to be used across various sectors, from agriculture and forestry to veterinary and public health. For example, Neem oil (*Azadirachta indica*) contains azadirachtin, which functions as an insect growth regulator and antifeedant. Studies show it effectively controls pests like aphids, whiteflies, and caterpillars as well as mosquitoes [105,106,107,108]. Citronella oil (*Cymbopogon citratus*; *C. nardus*), derived from lemongrass, is well known for repelling mosquitoes and other insects [109,110]. Studies highlight its significant effectiveness as a natural mosquito repellent. Tobacco leaf extracts (*Nicotiana tabacum*) have been effective against mosquito larvae, particularly *Aedes aegypti* [111]. In addition, tobacco extracts have shown high toxicity to pests like the rice weevil (*Sitophilus oryzae*) [112] and the cabbage aphid (*Brevicoryne brassicae*) [108]. For instance, aqueous extracts of *N. tabacum* have controlled aphid populations comparably or even better than synthetic pesticides, while being safer for natural predators.

##### Mechanism of Action of PEO and PE Controlling *A. diaperinus*

PEO and PE target ion channels and disrupt neurotransmitter signaling in insects. By inhibiting the flow of ions such as sodium and potassium, these compounds cause neural dysfunction, paralysis, and eventual mortality. Studies confirm their efficacy in blocking voltage-gated sodium channels, mimicking the action of synthetic insecticides while reducing environmental impact [113,114].

The lipophilic nature of PEO enables them to penetrate the hydrophobic insect cuticle. This disrupts the lipid layer that preserves moisture, leading to dehydration and desiccation. Research highlights this as a primary mode of action for many essential oils, contributing significantly to their insecticidal properties. PEO with antimicrobial properties suppress symbiotic gut bacteria in insects like *A. diaperinus*. These bacteria are vital for digestion and nutrient absorption. By disrupting microbial communities, PEO cause malnutrition and starvation in target pests [115].

Certain plant-derived compounds inhibit juvenile hormone and ecdysone, two key regulators of insect growth and reproduction. For example, eugenol has been shown to disrupt juvenile hormone pathways, impairing molting and reproductive cycles in pests. This hormonal imbalance leads to reduced fertility and developmental failure, effectively controlling insect populations. Phenolic compounds such as eugenol and cinnamaldehyde generate reactive oxygen species in insect cells. These reactive oxygen species induce oxidative stress, which damages cellular components like proteins, lipids, and DNA. Over time, this oxidative damage overwhelms the insect’s antioxidant defenses, leading to cell death [116].

PEO constituents interfere with cellular respiration by impairing mitochondrial function. This leads to reduced ATP production, a critical energy source for cellular processes. The accumulation of volatile compounds in insects with small spiracular openings, such as *A. diaperinus*, exacerbates this effect, resulting in energy depletion and death [117].

##### Studies on the Control of *A. diaperinus* with POE and PE

In pest control, PEO can disrupt the life cycles of various pests by acting as insecticides, repellents, and growth inhibitors. They are especially effective against larvae, reducing pest populations before they can reach adulthood and reproduce. This is particularly relevant for managing pests like the lesser mealworm, a major concern in poultry farming. Arena et al. [118] explored the repellent properties of several PEO, including clove (*Syzygium aromaticum*), oregano (*Origanum vulgare*), and *Dysphania ambrosioides*. These oils demonstrated strong repellent effects, with up to 95% of adult mealworms avoiding treated areas within the first two hours. When added to poultry feed, *O. vulgare* and *S. aromaticum* retained their repellent activity, preventing up to 78% of the insects from consuming treated feed within 24 h. These findings suggest that incorporating essential oils into poultry feed or litter could be a valuable strategy for controlling *A. diaperinus* in poultry houses. Despite their general effectiveness, not all PEO perform equally well against *A. diaperinus*. Velusamy et al. [119] tested PEO from eucalyptus (*Eucalyptus globulus*) and lemon grass (*Cymbopogon citratus*) and found that even at high concentrations (20%), these oils did not cause significant mortality in larvae or adult beetles. This suggests that certain PEO, while possessing repellent properties, may not be effective as direct insecticides against lesser mealworm.

Studies have shown that PEO from different plants, such as thyme (*Thymus vulgaris*) and neem (*Azadirachta indica*), are effective in controlling the lesser mealworm. These oils exhibit both larvicidal and adulticidal effects, as well as repellent properties that deter egg-laying and reduce larval development. For instance, Santana et al. [120] found that PEO from *Myrcia oblongata* had insecticidal properties with mortality rates exceeding 95% in both larvae and adult stages of *A. diaperinus*. The study also highlighted the antimicrobial effects of these oils against pathogens like *Salmonella*, indicating their potential dual role in controlling both pests and harmful bacteria in poultry environments.

In one study, it was found that *Cunila angustifolia* oil effectively reduced larvae and adult of *A. diaperinus* populations both in vitro and in vivo. At 5% and 10% concentrations, the oil achieved 100% mortality in vitro, while in vivo, a 5% concentration applied twice showed the best results. These findings suggest *C. angustifolia* oil as a promising alternative for controlling lesser mealworms in poultry facilities [121].

In a study *Ricinus communis* (castor plant) PE applied at an 8% concentration showed the highest mortality rate at 59.0%, while *Chenopodium ambrosioides* (Mexican tea) demonstrated moderate efficacy with a 44.6% mortality rate for *A. diaperinus*. In contrast, *Baccharis trimera* (carqueja) showed no significant insecticidal effect. These findings suggest that *R. communis* and *C. ambrosioides* hold promise as bio-insecticides [122].

Szczepanik et al. [123] tested *Origanum vulgare* and *Artemisia dracunculus* PEOs for their insecticidal and antimicrobial effects. They found that oregano oil, rich in carvacrol, significantly inhibited the growth of *A. diaperinus* larvae, reducing body weight and overall development. In contrast, tarragon oil, with methyleugenol as its main component, showed much lower insecticidal activity. The antimicrobial properties of oregano oil against bacteria such as *Escherichia coli* and *Staphylococcus aureus* were also notable, further supporting its use as a multifunctional agent in poultry farming.

A study investigated the impact of starch granules enriched with carvacrol (3%, 5%, and 10%) mixed with straw pellets as poultry litter on the mortality of *A. diaperinus* and the growth performance of broiler chickens exposed to the treated litter [124]. In bioassays simulating chicken house conditions, granules and pellets were mixed in ratios of 30:70%, 40:60%, and 50:50% and tested against young larvae, older larvae, and adult mealworms at 29 °C in the dark. The most effective treatment was 10% carvacrol-enriched granules at a 40:60% ratio, which killed all larvae and adults within 3–4 days. Broiler chickens raised on treated litter showed similar growth performance to the control group, with a slightly higher feed conversion rate (1.72 vs. 1.56) and a non-significant reduction in final body weight (100 g less than the control). This treatment appears promising for controlling *A. diaperinus* in broiler houses.

Pinto Junior et al. [125] evaluated the insecticidal effects of sassafras (*Ocotea odorifera*) and eucalyptus (*Eucalyptus viminalis*) PEO on larvae and adults of *A. diaperinus*. Larvae were more susceptible to sassafras oil but less susceptible than adults to eucalyptus oil. Sassafras oil showed higher efficacy (LC_50_: 0.12 mL/L for larvae and 0.26 mL/L for adults) compared to eucalyptus oil (LC_50_: 1.72 mL/L for larvae and 1.37 mL/L for adults). These results suggest that both essential oils, particularly sassafras, have potential for use in integrated pest management strategies against lesser mealworms.

The insecticidal and antifeedant effects of *Illicium verum* (star anise) fruit extracts on *A. diaperinus* were evaluated, revealing that larvae were more susceptible than adults. Among the larvae, 7-day-old individuals were the most sensitive, while 30-day-old larvae showed high tolerance [126]. All tested concentrations (3.12–25.0 mg/mL) caused 100% mortality in younger larvae, while only higher concentrations (12.5 and 25.0 mg/mL) were effective against older larvae. Adults showed no mortality even at the highest concentration. The extract significantly deterred feeding in larvae in choice tests but had weak antifeedant effects in no-choice tests. Lower concentrations allowed some larvae to develop but resulted in reduced body weight compared to controls.

The evaluation of *Illicium verum* (star anise) PEO revealed its insecticidal effects on *A. diaperinus*, particularly in terms of survival, biochemical parameters, and locomotor behavior [127]. Sublethal concentrations (0.5% and 1%) did not increase reactive oxygen species but affected key enzymes: 0.5% increased glutathione s-transferase activity, while both concentrations reduced acetylcholinesterase activity. Behavioral changes included loss of refuge-seeking at 0.5% and impaired locomotion at 1%. These findings suggest that *I. verum* essential oil could be a viable alternative to conventional insecticides for controlling lesser mealworms.

On the other hand, in a study under controlled lab conditions, treatments included aqueous and hydroalcoholic PE (*Cinnamomum verum* L., *Allamanda cathartica* L., *Ateleia glazioveana* Baill, *Cymbopogon* sp., *Chrysantemum* sp.), neem-based commercial products, and synthetic insecticides (lambda-cyhalothrin and dichlorvos). Dichlorvos and Neem achieved 100% efficiency against adult mealworm, while lambda-cyhalothrin and all PE showed no insecticidal activity [128].

One of the major advantages of using PEO and PE in pest control is their environmental safety. These natural products degrade quickly, leaving no harmful residues, which is particularly important in agricultural and poultry farming environments. PEO, such as those derived from *Eucalyptus citriodora* [129], have been shown to reduce *A. diaperinus* populations in poultry farms by up to 91%, without negatively affecting the health or growth of chickens. This makes essential oils a sustainable alternative to chemical insecticides, which can have long-lasting negative effects on both the environment and non-target organisms. However, comprehensive studies, including toxicity assessments, formulation development, and field trials, are necessary to evaluate their safety for poultry, workers, and the environment, as well as their practical application.

##### Synergistic Effect Studies with PEO and PE on *A. diaperinus* Control

The combination of different PEO can enhance their overall efficacy, often showing a synergistic effect. Francikowski et al. [130] evaluated various essential oils, including mint, vanilla, lemon, and citronella, and found that mixtures of oils, particularly a 1:1 combination of lemon and vanilla, had a stronger repellent effect than the individual oils. Citronella oil was highly effective, with strong repellency at both low and high concentrations (1% and 10%).

The combined effects of neem and citronella oils on *A. diaperinus* were investigated, revealing that citronella oil at a 20% concentration achieved 81% mortality after 10 days, while neem oil at 9% resulted in 59% mortality [131]. When combined, the oils exhibited an additive effect, further enhancing their insecticidal properties. These findings highlight the potential of using PEO mixtures as effective alternatives to synthetic insecticides.

In addition to pure PEO, PE and innovative formulations such as nanoemulsions have been studied for their potential in pest control. The larvicidal and adulticidal effects of *Cinnamomum zeylanicum* (cinnamon) essential oil were tested, both in its pure form and as nanostructured formulations. At a 10% concentration, pure cinnamon oil achieved 100% mortality in larvae and adult beetles within 11 days [132]. Nanoemulsions and nanocapsules of cinnamon oil also showed high efficacy, though slightly lower than the pure form, with mortality rates reaching 80% for larvae and 85% for adults. These results suggest that nanoencapsulation of PEO could reduce environmental toxicity while maintaining insecticidal properties.

The compatibility of PEO from *Mentha* species with the entomopathogenic fungus *B. bassiana* was investigated. Essential oils from *Mentha arvensis*, *Mentha spicata*, and *Mentha piperita* were found to be highly toxic to *A. diaperinus* larvae while remaining compatible with fungal biocontrol agents. These findings suggest that PEO can be incorporated into IPM programs without reducing the effectiveness of other biological control methods [133].

#### 3.2.2. Pheromones

Pheromones (P) are chemical signals used by insects to communicate with individuals of the same species, making them an essential tool in pest control, especially for managing insect populations. These chemical compounds play a crucial role in regulating important behaviors like reproduction, feeding, and defense. By mimicking or interfering with these natural signals, pheromones can be used to manipulate pest behavior, including attraction to traps or disruption of mating processes, leading to population reduction.

P are released from specialized glands in insects and can travel considerable distances—sometimes up to 2–3 km—affecting other individuals. Despite being released in minuscule quantities, even trace amounts can trigger strong behavioral responses, such as attraction, mating, or dispersal. One of the most valuable aspects of pheromone-based pest control is that it targets specific species without harming non-target organisms, and it doesn’t lead to residue buildup, which makes pheromones an environmentally friendly pest control option.

Studies have extensively explored the use of P in pest control, focusing on their ability to manipulate insect behavior in environmentally friendly ways. For instance, synthetic sex P have been utilized to disrupt mating patterns in pest populations, a technique known as mating disruption. Research on codling moths (*Cydia pomonella*), a significant pest in apple orchards, demonstrated that the release of synthetic P significantly reduced mating success and larval infestation rates, making it an effective alternative to chemical pesticides [134]. Similarly, aggregation P have been used to attract pests to traps, such as bark beetles in forestry management, where this approach minimizes collateral damage to non-target species [135]. These studies highlight the potential of P to provide sustainable and targeted pest control solutions.

##### Mechanism of Action of P Controlling *A. diaperinus*

Male beetles produce aggregation P, which attract both males and females to specific areas, aiding in their congregation and facilitating targeted pest control strategies. These P are chemically complex, with specific blends and ratios determining their effectiveness in luring beetles to traps. Insects are attracted to traps using these chemicals. Alarm Ps have also been identified, which can disperse insect populations into critical areas and provide an additional method for behavior-based pest management [2]. Alarm P can also be used to achieve insect control using a “push-pull” system. In this system, aggregation P (which attracts pests) are used in combination with alarm P (which repel pests) to create a dual-action approach [136].

Additionally, pheromone-based traps can be integrated with biological control agents, such as *B. bassiana*, a fungal pathogen. This combination enhances pest management by simultaneously trapping beetles and inoculating them with pathogenic spores, leading to colony-wide impacts [137].

##### Studies on the Control of *A. diaperinus* with P

Research has shown that male lesser mealworm release a six-component aggregation P, consisting of (R)-limonene, (E)-ocimene, 2-nonanone, (S)-linalool, (R)-daucene, and (E,E)-α-farnesene. These compounds have been identified as potent attractants, especially when combined in specific ratios, and are effective in trapping both larvae and adult beetles, making them valuable in IPM strategies.

In a study, Bartelt et al. [138] identified the male-produced aggregation P of *A. diaperinus* and confirmed its ability to attract both male and female beetles. A synthetic blend of these pheromones was tested in poultry production facilities, successfully capturing both sexes, demonstrating the efficacy of P traps in controlling lesser mealworm populations. This research highlights the potential of using P-based strategies to reduce pest populations in poultry houses without relying on chemical insecticides.

Interestingly, the composition of aggregation P can vary between geographic populations. Hassemer et al. [139] revisited the P composition of a Brazilian population of *A. diaperinus* and identified six key components: (R)-limonene, (E)-ocimene, 2-nonanone, (S)-linalool, (R)-daucene, and (E,E)-α-farnesene. While five of these components had been previously identified in American populations, the sixth component, (E,E)-α-farnesene, was unique to the Brazilian population. This discovery underscores the importance of understanding regional variations in P composition for developing effective pest management strategies.

Research was conducted on developing a push-pull system for managing *A. diaperinus* in poultry houses. The study tested synthetic aggregation P (including (R)-limonene, 2-nonanone, (E)-ocimene, and others) as well as alarm P (1,4-benzoquinone and its derivatives) [136]. In laboratory and field tests, aggregation P successfully lured beetles into traps, while alarm P repelled them from certain areas. This combination enhanced capture rates and provided an eco-friendly alternative to insecticides.

##### Synergistic Effect Studies with P on *A. diaperinus* Control

One of the challenges of using P in pest management is ensuring their sustained release over time. Vaz Júnior et al. [140] explored the development of an organic-inorganic composite from calcium carbonate and Kraft lignin as a carrier material for the controlled release of limonene, a key component of the lesser mealworm’s aggregation P. The study showed that the composite materials allowed for the slow release of limonene over 30 days, indicating the potential for long-lasting P traps in pest management systems. This slow-release technology could improve the effectiveness of P-based traps, reducing the need for frequent reapplication and maintaining consistent pest control.

The attractiveness of an aggregation P lure combined with chicken droppings to *A. diaperinus* adults and larvae was evaluated. Field tests showed that P-baited traps captured significantly more beetles compared to controls, highlighting the potential of P in IPM programs for effectively managing lesser mealworm populations [141].

#### 3.2.3. Diatomaceous Earth

Diatomaceous earth (DE) is a naturally occurring, non-toxic powder composed of fossilized remains of diatoms, a type of hard-shelled algae. It has gained significant attention in IPM due to its environmentally friendly nature and effectiveness in controlling various pests. This makes DE a promising alternative to chemical insecticides, particularly in poultry farming where pests like *A. diaperinus* cause economic losses by damaging poultry house structures and spreading pathogens.

Research has shown its effectiveness against stored-product pests, such as the red flour beetle (*Tribolium castaneum*), lesser grain borer (*Rhyzopertha dominica*), rice weevil (*Sitophilus oryzae*) [142,143]. For instance, Athanassiou et al. [143] demonstrated that DE-treated grains significantly reduced insect survival rates, with efficacy influenced by dose, exposure time, and humidity levels. Similarly, Fields and Korunic [144] highlighted its potential as a sustainable alternative to chemical pesticides, emphasizing its compatibility with integrated pest management strategies. These findings underscore DE’s versatility as an environmentally safe tool for pest control in agriculture and storage systems.

##### Mechanism of Action of DE Controlling *A. diaperinus*

The primary advantage of DE over chemical insecticides lies in its mode of action. DE is not toxic to humans, animals, or non-target organisms, making it safe for use in environments where food is produced, such as poultry houses. It causes physical damage to the exoskeletons of insects, leading to dehydration, and as insects cannot develop resistance to this mechanical action, DE remains effective over long periods. DE works primarily through a mechanical action; when insects come into contact with DE, the powder adheres to their exoskeleton, absorbing oils and fats, which leads to desiccation and ultimately, death. However, its effectiveness depends on environmental conditions such as humidity and substrate type, as the powder needs to stay dry to function properly [145].

##### Studies on the Control of *A. diaperinus* with DE

Several studies have demonstrated the efficacy of DE in controlling lesser mealworm populations under various conditions. Oliveira et al. [146] confirmed DE’s efficacy in reducing *A. diaperinus* populations by 80% when applied at a concentration of 280 g/m^2^. The study emphasized that for maximum efficiency DE should be reapplied before every new lot of birds, specifically in high-infestation zones like under feeders and near walls and pillars. The use of DE was comparable to chemical treatments in reducing pest populations, supporting its use as a sustainable option in IPM programs.

DE’s long-term effectiveness has also been evaluated in various studies. Oliveira et al. [147] assessed the persistence of DE in poultry litter over eight months and found that even after this period, DE retained 60% of its insecticidal activity. Additionally, DE exhibited strong repellency against *A. diaperinus*, causing significant reductions in insect captures in treated areas compared to non-treated areas. This repellency can be beneficial in directing pests away from critical areas like feedlines or egg-laying zones.

Similarly, the effectiveness of DE, spinosad, and cyfluthrin on broiler farms in southeastern Queensland was studied by Lambkin et al. [148]. They reported that while spinosad outperformed DE on hard floors, DE still showed promise in controlling lesser mealworms when applied to certain areas like under feeders or walls. However, in some instances, the efficacy of DE was not significantly better than untreated controls, particularly when environmental conditions (such as moisture) were not optimal for DE’s mode of action.

Agrafioti et al. [149] demonstrated that the effectiveness of DE formulations varies depending on the insect species and environmental conditions. In the case of *A. diaperinus*, the most effective formulation, Silicid, achieved 100% mortality within 21 days at the highest dose rate. However, other DE formulations, such as Celatom^®^ MN-23 and SilicoSec^®^, had significantly lower efficacy, indicating that not all DE products are equally effective, and careful selection of the formulation is critical.

A study also explored the factors influencing the effectiveness of DE, including temperature, substrate type, and its repellency to adult beetles. The findings revealed that DE was more effective at higher temperatures (32 °C) and performed best in dry, clean substrates such as chicken feed, where mortality rates reached 95% [150]. In contrast, its efficacy was significantly reduced in poultry litter, likely due to the litter absorbing and removing DE particles. This highlights the importance of applying DE in areas of the poultry house that are less prone to moisture, ensuring optimal pest control results.

While DE is a promising tool in pest management, several factors can affect its performance. High humidity levels or damp conditions can reduce DE’s efficacy because moisture prevents the powder from adhering to the insects’ exoskeletons. Therefore, it is essential to ensure that DE is applied in dry areas or combined with other pest control methods that are more effective in humid environments. Additionally, the powder’s mechanical action requires direct contact with insects, so strategic application is necessary to ensure that *A. diaperinus* encounters the treated surfaces.

##### Synergistic Effect Studies with DE on *A. diaperinus* Control

Santoro et al. [151] evaluated the combination of *B. bassiana* and DE for controlling *A. diaperinus*. They found that the combination of these two agents produced higher mortality rates than either treatment alone, demonstrating a synergistic effect. However, the study also noted that the presence of effective microorganisms reduced the overall effectiveness of the fungal component, suggesting that careful consideration of biological interactions is necessary in IPM programs.

The various biological control and sustainable control agents outlined above have proven to be valuable tools for managing the lesser mealworm. All these methods are summarized in Table 1.

## 4. Conclusions

EF particularly *B. bassiana* and *M. anisopliae*, show significant promise as biological control agents against the lesser mealworm, especially in targeting larvae. While adult beetles are generally more resistant to fungal infections, larvae are highly susceptible, with some fungal strains achieving 100% mortality. Combining fungi with insecticides or using fungal extracts can further enhance their efficacy. Environmental conditions, such as temperature and poultry litter, play a crucial role in determining fungal performance, and these factors must be considered in integrated pest management strategies. Overall, fungi-based biopesticides offer a sustainable and effective alternative to chemical insecticides for managing *A. diaperinus* populations in poultry facilities.

While EB like *B. thuringiensis* are highly effective against many agricultural and forestry pests, their efficacy against *A. diaperinus* remains limited. Studies have shown variability in results, with some promising outcomes in early larval stages but less success against more developed larvae and pupae. Enhancing the toxicity of *Bt* toxins with synergists like cadherin fragments may offer a way to improve control efficacy against *A. diaperinus*. Overall, more research is needed to optimize bacterial strains and application methods for effective control of this pest in poultry facilities.

EN are a cornerstone of biological control. Species such as *S. carpocapsae*, *S. feltiae*, and *H. bacteriophora* have shown promising results in controlling lesser mealworm populations, especially at the larval stage. Nematodes invade the host through natural body openings and release symbiotic bacteria that rapidly kill the insect. Studies have highlighted the superior susceptibility of *A. diaperinus* larvae to nematodes due to their thin cuticles and higher mobility, which increase their contact with the nematodes. While adult beetles, with their thicker cuticles, are more resistant, nematodes remain a powerful option, particularly when integrated with other biological agents for comprehensive control.

PEO and PE present a promising and sustainable alternative to synthetic pesticides for managing lesser mealworm populations in poultry farms. Their insecticidal, repellent, and antimicrobial properties make them versatile tools in integrated pest management strategies. Essential oils such as thyme, neem, oregano, and cinnamon have demonstrated significant efficacy in both laboratory and field trials, especially against larvae. Moreover, the use of oil combinations and advanced formulations, such as nanoemulsions, can enhance their insecticidal potential while reducing environmental risks. As research continues to explore the full potential of plant-based pest control methods, essential oils are likely to play a key role in the future of sustainable agriculture and poultry management.

P offer an environmentally friendly and efficient solution for controlling pest populations, including the lesser mealworm. Their ability to influence insect behavior at minimal concentrations, along with their species-specificity, makes pheromones an ideal tool for integrated pest management strategies. By combining aggregation pheromones with alarm pheromones or other control methods, pest management systems can achieve higher capture rates and more effective control over hidden or hard-to-reach populations. Advances in slow-release pheromone dispensers further enhance the long-term potential of pheromone traps in sustainable pest management efforts.

DE is an environmentally friendly and effective alternative to chemical insecticides for controlling lesser mealworm populations in poultry farms. Its physical mode of action ensures that pests cannot develop resistance, making it a long-term solution for pest control. However, its efficacy can be influenced by environmental factors like humidity and substrate type, which must be managed for optimal results. When combined with other control agents such as *B. bassiana* or used as part of a broader IPM strategy, DE can play a crucial role in sustainable pest management programs aimed at reducing *A. diaperinus* populations in poultry environments.

In conclusion, biological control strategies, including the use of EF, EB, EN, PEO and PE, P and DE, are proving to be effective alternatives to traditional chemical methods. These agents not only reduce pest populations but also address concerns about environmental impact and insecticide resistance. The integration of these biological tools into IPM programs offers a sustainable and effective solution for managing *A. diaperinus* in poultry environments.

## Figures and Tables

**Table 1:** Biological and sustainable control agents for managing lesser mealworm populations: types, effective species, and mechanisms of action.

**Types of Biological Control Agents**	**Some Effective Species**	**Summary of Action Mechanism**
Entomopathogenic fungi (EF)	*Beauveria bassiana* *Isaria farinosa* *I. fumosorosea* *Metarhizium anisopliae*	Entomopathogenic fungi infect insect hosts by directly penetrating their cuticle using enzymatic and mechanical mechanisms, proliferating within the host, producing toxic metabolites, and eventually sporulating to continue their life cycle.
Entomopathogenic bacteria (EB)	*Bacillus thuringiensis*	Entomopathogenic bacteria kill insect hosts by producing toxins that either attack gut cells or suppress the immune system, leading to septicemia and death, with some bacteria, like *Bacillus thuringiensis* acting through gut disruption and others, like *Photorhabdus* and *Xenorhabdus*, working in symbiosis with nematodes.
Entomopathogenic nematodes (EN)	*Steinernema carpocapsae* *S. affine* *S. affinis* *S. feltiae* *Heterorhabditis bacteriophora* *H. riobrave* *H. indica*	Entomopathogenic nematodes kill their insect hosts either by releasing symbiotic bacteria that cause toxic effects or by direct mechanical damage and nutrient depletion, thriving in moist environments where they invade through natural openings, reproduce within the host, and effectively control pests without leaving harmful residues or inducing resistance.
**Types of sustainable control agents**	**Some effective compounds**	**Summary of action mechanism**
Plant essential oils (PEO) Extracts (PE)	*Azadirachta indica* oil *Cymbopogon citratus* oil *Origanum vulgare* oil *Syzygium aromaticum* oil *Ricinus communis* extract *Illicium verum* extract *Cinnamomum verum* extract	Plant essential oils and plant extracts exert insecticidal effects by disrupting ion channels and neurotransmitter signaling, damaging the cuticle, suppressing gut bacteria, interfering with hormonal regulation, inducing oxidative stress, and impairing mitochondrial function, ultimately leading to insect mortality.
Pheromones (P)	(E,E)-α-farnesene (S)-linalool (R)-daucene (R)-limonene 2-nonanone (E)-ocimene 1,4-benzoquinone	Pheromones, including aggregation and alarm types, are used to attract or repel male and female beetles, enabling behavior-based pest control strategies such as “push-pull” systems and integration with biological agents like *Beauveria bassiana* for enhanced management.
Diatomaceous earth (DE)	Silica cell walls of ancient diatoms	Diatomaceous earth is a non-toxic, long-lasting insect control method that physically damages insect exoskeletons, causing dehydration without the risk of resistance, but its effectiveness depends on environmental conditions like humidity and substrate type.

## Data Availability

No new data created in this manuscript.

## Abbreviations

The following abbreviations are used in this manuscript:

*Bt*	*Bacillus thuringiensis*
Cry	Crystalline
DE	Diatomaceous Earth
EB	Entomopathogenic Bacteria
EF	Entomopathogenic Fungi
EN	Entomopathogenic Nematodes
IPM	Integrated Pest Management
P	Pheromones
PE	Plant Extracts
PEO	Plant Essential Oils
